# Factors affecting quality of life of people living with HIV/AIDS attending a community care center in Chennai, India

**DOI:** 10.1186/1471-2334-14-S2-P43

**Published:** 2014-05-23

**Authors:** G Shanmugasundaram Anusuya, NC Appavoo, T Mahalakshmy, AS Valan, PJ Parameaswari, PM Udayshankar, T Stephen

**Affiliations:** 1 Sree Balaji Medical College and Hospital, Bharath University, Chennai, India; 2 Department of Community Medicine, JIPMER, Pondicherry, India; 3 International Training and Education Center for Health, India

## Introduction

Health is defined by WHO as “complete physical, mental and social well-being”. Though the physical well-being is improved with antiretroviral therapy, it does not guarantee mental and social well-being. Hence we wanted to assess the Quality of Life (QOL) and to identify the factors that influences QOL in People Living with HIV/AIDS (PLHA)

## Materials and methods

A cross sectional study of 141 adult PLHA attending the out patient department of a community care center in Chennai during the period from 1st January to 31st August 2011 were included in the study.QOL was evaluated using WHOQOL-HIV BREF questionnaire. Analysis: Mean Scores of QOL was calculated using SPSS syntax file developed by WHO, Geneva. The One way Analysis of Variance (ANOVA) was performed to find out significant difference between the socio-demographic variables and clinical categories on QOL domains.

## Results

In study population 76 (53.9%) were males, 59 (41.8 %) were females and 6 (4.3 %) transgender. The overall QOL mean score on a scale of 0-20 was found to be 13.1. The mean scores of six domains of QOL in descending order were Spirituality/Religion/Personal beliefs (SRPB) (14.5); level of independence (LOI) (14.3); physical (13.3); environmental (12.5); psychological (12.3) and social relationships domain (11.7): (p-value=0.000). QOL in physical domain was high among transgenders (17.3) when compared with males (12.7), and females (13.7): (p=0.019). Social relationships domain scores between those who were single (11.5), married (12.8), and separated/widowed/divorced (10.9):(p=0.000). LOI domain was high among employed (14.5) than the unemployed (13.2): (p=0.028). Significantly better QOL scores in the LOI domain (15.2) (p=0.000) with respect to the CD4 count category. LOI domain better among patients without current history of tuberculosis (14.5) (p=0.008).

## Conclusions

In our study, QOL was associated with gender, marital status, employment status, CD4 counts of the patients and current history of tuberculosis.

